# P-1624. Are Follow-Up Blood Cultures Necessary in Children with Gram-Negative Rod Bacteremia?

**DOI:** 10.1093/ofid/ofae631.1791

**Published:** 2025-01-29

**Authors:** Shreya M Doshi, Shamily Jadhav, Maria Susana Rueda Altez, Emily Ansusinha, Rana F Hamdy, Rana F Hamdy

**Affiliations:** Children's National Health System, Washington DC, District of Columbia; George Washington University of Medicine and Health Sciences, Washington, District of Columbia; University of Alabama at Birmingham, Birmimgham, Alabama; Children's National Hospital, Washington, District of Columbia; Childrens National Hospital, Washington, District of Columbia; Childrens National Hospital, Washington, District of Columbia

## Abstract

**Background:**

The utility of follow-up blood cultures (FUBCs) in children with a gram-negative rod (GNR) bacteremia is unclear. A prior pediatric study showed that FUBC positivity rate was 21%. Many adult studies have demonstrated that a FUBC is not always necessary in the setting of a UTI. Primary objectives were to determine 1. the rate of positivity in follow up blood cultures in children with gram negative bacteremia 2. To understand the clinical risk factors associated with positivity in follow up blood cultures in children with gram-negative rod bacteremia.

**Figure 1:**
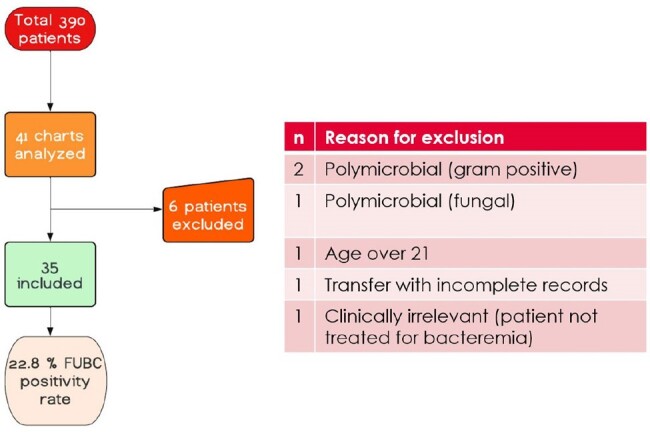
Flowchart depicting chart analysis

**Methods:**

A retrospective cohort study in patients < 21 years of age at Children’s National Hospital that had a positive blood culture for gram negative rods from 01/2013 to 06/2023. FUBC was defined as a subsequent blood culture collected at least 24 hours after and < 7 days from the time of collection of the initial positive blood culture. Structured chart review was performed to collect clinical features, source of bacteremia, time to positivity, and FUBC positivity and contamination rate . Univariate analysis of each risk factor with FUBC positivity and multi-variable logistic regression model for association of each risk factor with FUBC positivity will be performed. Figure 1 - Flowchart depicting chart analysis.

**Table 1.**
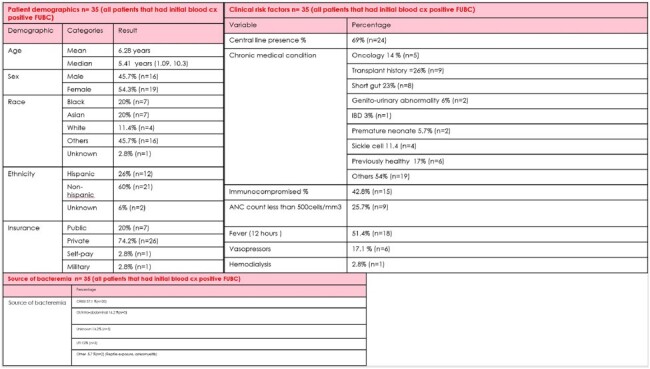
Demographics and Clinical Characteristics of patients

**Results:**

In an analysis of 35 patients, rate of FUBC positivity was 23% (n=9), contamination rate in the FUBC was 11% (n=1). Clinical factors of and outcomes are depicted in Table 1 and 2. *E.coli* was the most common organism found in the initial blood culture depicted in Figure 2.

**Table 2.**
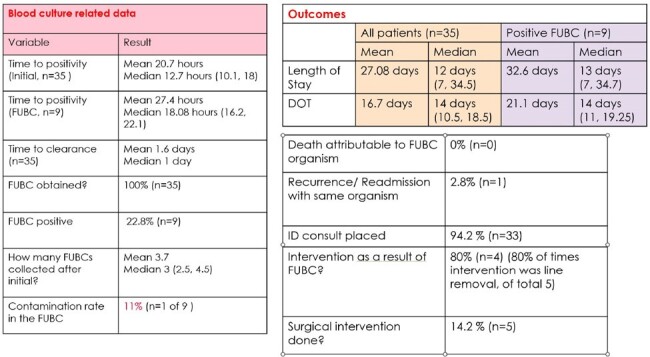
Blood culture data and outcomes

**Conclusion:**

Preliminary results for this ongoing study demonstrate that FUBC positivity was 22% in children with gram negative bacteremia, which is higher than what has been reported in adult studies. More data and subset analysis required for immunocompromised patients and patients with a central line.

**Figure 2:**
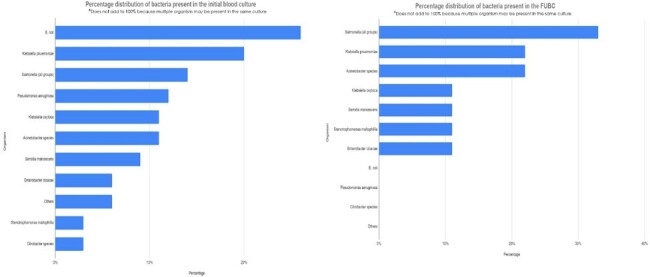
Microbiologic distribution by percentage for initial and follow-up blood culture

**Disclosures:**

**All Authors**: No reported disclosures

